# Rights-seeking, racism, and retribution

**DOI:** 10.1016/j.lanwpc.2025.101481

**Published:** 2025-01-24

**Authors:** Bronwyn Wilkes, Lisa J. Whop, Raglan Maddox, Katherine A. Thurber, Christopher D. McKay, Clinton Schultz, Carla McGrath, Olivia Evans, Justyce Pengilly, Mikala Sedgwick, Fiona Cornforth, Raymond Lovett

**Affiliations:** a Yardhura Walani, National Centre for Aboriginal and Torres Strait Islander Wellbeing Research, The Australian National University, Acton, ACT, 2601, Australia; b Black Dog Institute, Hospital Road, Prince of Wales Hospital, Randwick, NSW, 2031, Australia; c School of Medicine and Psychology, The Australian National University, Acton, ACT, 2601, Australia; d Carla McGrath Consulting, Minjerribah, QLD, 4183, Australia

## Rights-seeking, racism, & retribution

The right to the “highest attainable standard of physical and mental health” is embedded in the International Covenant on Economic, Social and Cultural Rights.^1^ For Indigenous Peoples, this right is realised through self-determination and freedom from colonial structures. The right to self-determination is central to international human rights apparatus, including the United Nations Declaration on the Rights of Indigenous Peoples, which recognises the right of Indigenous Peoples to “be free from any kind of discrimination, in the exercise of their rights.”^2^ Self-determination for Aboriginal and Torres Strait Islander peoples continues to be challenged and denied by settler-colonial systems and practices that perpetuate structural violence and cause systemic inequities by preventing access to political, social, and cultural determinants of health.

Structural change, rooted in human rights and justice, is essential to dismantle these settler-colonial systems and enable Aboriginal and Torres Strait Islander peoples to reclaim the determinants of health and wellbeing. However, calls for justice and the pursuit of structural change are often met with (re)active racism, resistance, and retribution as dominant systems and groups seek to maintain and entrench power.^3^^,^^4^ This has been evident in the lead up to and aftermath of the failed referendum on 14 October 2023, which sought to embed a mechanism within the Australian constitution for Aboriginal and Torres Strait Islander peoples to provide advice to government about policies affecting their lives. Leading up to the referendum, Aboriginal and Torres Strait Islander adults experienced substantial increases in interpersonal discrimination and racism, including in healthcare settings, and substantial declines in numerous aspects of wellbeing.^5^ Discrimination and racism continue to be pervasive and elevated 12-months since the referendum, and the visceral effects of this are evident in many aspects of wellbeing that remain worse than levels seen in years preceding the referendum.^5^

The rejection of the referendum signalled a reinforcement of settler-colonial privilege and emboldened those seeking to uphold oppressive systems. Examples of amplified structural racism were evident soon after the referendum, including in retractions from treaty and truth-telling processes. In Queensland, where the ‘no’ vote was 68%, the incoming Government repealed the *Path to Treaty Act 2023 (QLD)* and abolished Queensland's Truth-telling and Healing Inquiry. This Act supported the development of a framework for treaty negotiations and established the Truth-telling and Healing Inquiry designed to report on the impacts of colonisation on Aboriginal peoples and Torres Strait Islander peoples, and help heal the trauma suffered as a result of colonisation.^6^ Further examples of active resistance to Indigenous self-determination and human rights include regressive youth crime legislation introduced in Queensland and Northern Territory. These policies disproportionately target Aboriginal and Torres Strait Islander children, despite evidence that the measures are harmful and ineffective.^7^ The Northern Territory Government's decision to lower the age of criminal responsibility from 12 to 10 years—a move that will almost exclusively affect Aboriginal and Torres Strait Islander children^8^—contravenes obligations under the United Nations Convention on the Rights of the Child and directly opposes recommendations of the Northern Territory Royal Commission into the Protection and Detention of Children.^9^ The Queensland Government's introduction of “adult crime, adult time” under the *Making Queensland Safer Bill 2024*, egregiously required overriding the state's own human rights legislation given the incompatibility of the policy with human rights.^10^ These are contemporary examples of settler-colonial tactics of dehumanising Indigenous peoples, denying Indigenous rights, and silencing truth telling. Such measures perpetuate intergenerational harms, compounding the health and wellbeing challenges faced by Aboriginal and Torres Strait Islander peoples and communities.

These actions reflect more than the aftermath of a rejected referendum—they are part of a systemic escalation of the oppressive power of settler-colonial structures. Aboriginal and Torres Strait Islander peoples are burdened with the dual task of resisting these systems while simultaneously enduring their harms. This dynamic entrenches a vicious cycle of inequity that prioritises settler-colonial privileges over justice, healing, and humanity. These trends are not unique to Australia but reflect a broader geopolitical climate in which efforts to address historical and ongoing structural injustices are being undermined by the rise of nationalist, populist, and conservative forces that seek to maintain colonial power.^11^ For example, recent policy changes in Aotearoa New Zealand have abolished Te Aka Whai Ora (the Māori Health Authority) and wound back Māori rights in other areas; additional proposals threaten the cultural wellbeing of Māori children and diminish the rights recognised under Te Tiriti (the Treaty of Waitangi).^12^ This shared context underscores the urgency of collective action to dismantle these harmful systems to uphold justice and human rights.

The health, wellbeing, and prosperity enjoyed by non-Indigenous Australians are predicated on the dispossession of Aboriginal and Torres Strait Islander peoples. This disparity is a matter of unrealised justice, wherein settler-colonial systems continue to deny the full attainment of health for Indigenous peoples. As emphasised in an Editorial in *The Lancet*: “Racism is not only about the health of particular persecuted or excluded groups—it inflicts a collective trauma on us all. The positive corollary of this fact is that lessening inequities and restoring justice can bring healing to society as a whole.”^13^ True justice demands the dismantling of all forms of racism. Only when racism is eliminated will the highest attainable standards of health be realised for *all* peoples, Indigenous and non-Indigenous alike. This is not simply a matter of public health; it is an urgent call for humanity, justice, and an unwavering commitment to upholding human rights.

## Contributors

Conceptualisation: RL, KT, BW, CMcG, LJW, RM; Writing—original draft: BW, RL, KT; Writing—review & editing: BW, LJW, RM, RL, KT, CMcK, CS, OE, JP, CMcG, MS, FC.

## Declaration of interests

The Commonwealth Department of Health and Aged Care provided funding for a project about mental health and wellbeing in the lead up to and following the Voice to Parliament Referendum; KT, RL, BW, MS, CMcK, and OE are investigators on this project. This funding does not relate to this specific paper; this paper is not funded by or endorsed or approved by the Department of Health and Aged Care. It is conducted independently from our contract with the Department. CMcG is a member of the Thiitu Tharrmay Aboriginal and Torres Strait Islander Research Reference Group. Prior to involvement in this paper, FC was a member of the Australian Government's Referendum Engagement Group in her capacity as CEO of The Healing Foundation, and was Chair of the Project Advisory Group for First Nations Consultations conducted by First Nations Co for the Australian Human Rights Commission's Anti-Racism Framework.
